# Dynamic changes in macrophage polarization during the resolution phase of periodontal disease

**DOI:** 10.1002/iid3.70044

**Published:** 2024-10-22

**Authors:** Juhi R. Uttamani, Varun Kulkarni, Araceli Valverde, Raza Ali Naqvi, Thomas Van Dyke, Salvador Nares, Afsar R. Naqvi

**Affiliations:** ^1^ Department of Periodontics, College of Dentistry University of Iowa Iowa City Iowa USA; ^2^ Private Practice Austin Texas USA; ^3^ Department of Periodontics, College of Dentistry University of Illinois Chicago Chicago Illinois USA; ^4^ Department of Applied Oral Sciences The Forsyth Institute Cambridge Massachusetts USA; ^5^ Center for Clinical and Translational Research The Forsyth Institute Cambridge Massachusetts USA; ^6^ Department of Oral Medicine, Infection and Immunity Harvard School of Dental Medicine Boston Massachusetts USA; ^7^ Department of Microbiology and Immunology, College of Medicine University of Illinois Chicago Chicago Illinois USA

**Keywords:** macrophage, nonsurgical periodontal therapy, periodontal disease, polarization, resolution of inflammation

## Abstract

**Aim:**

Polarization of macrophages (Mφ) is a well‐controlled axis with considerable consequences in both the pro‐inflammatory and resolution phases of inflammation. We aimed to determine if periodontal therapy may instigate M1 to M2 Mφ polarization favoring resolution of inflammation within periodontal tissues.

**Methods:**

Gingival biopsies were excised from subjects diagnosed with Stage III, Grade B periodontitis before and 4–6 weeks after nonsurgical periodontal therapy. Total RNA was isolated and pro‐ and anti‐inflammatory markers associated with Mφ polarization assessed by RT‐qPCR. Mice were subject to ligature‐induced periodontitis and gingival tissues collected after 8 days in‐situ or 10 days after ligature removal and M1 and M2 Mφ markers examined by RT‐qPCR and flow cytometry.

**Results:**

In human samples, improvement in clinical parameters posttherapy correlates with reduced bacterial burden, downregulation in M1 (TNF‐α, STAT1, CXCL10, and miR‐155), and elevated levels of M2 (STAT6, TGM2, CCL22, and IL‐10) Mφ markers. In a murine model of resolution of LIP, we observed reduced levels of M1 Mφ markers *cox2*, *iNOS2*, F4/80+CD80+, and F4/80+CD86+ and elevated levels of M2‐like Mφ markers *tgm2*, *arg1*, F4/80+CD206+ and F4/80+CD163+ corroborated human findings.

**Conclusion:**

Resolution of periodontal inflammation is associated with M1 to M2 Mφ polarization after nonsurgical periodontal therapy. Assessment of Mφ markers can provide relevant clinical information on the successful response of periodontal therapy and may be used to target nonresponders.

## Graphical abstract

This is the first translational studies to longitudinally assess the M/M2 MΦ profiles before and after therapy in periodontitis subjects and can serve as the first step in examining current treatment strategies to include investigations on the role of macrophage immune mediated regulatory networks governing periodontal disease pathogenesis. Our findings highlight the immuno‐modulatory role of M1/M2 MΦ polarization in the periodontal disease pathogenesis and likewise, the impact of periodontal therapy in causing a switch toward M2 MΦ phenotype. Assessment of M2 macrophage markers may provide clinically relevant information to evaluate patient response to periodontal therapy and may be useful to target nonresponders with exaggerated immune responses.

## 1 INTRODUCTION

The classic chronic inflammatory state in periodontal disease is characterized by microbially induced molecular and cellular events fostering inflammation and loss of periodontal support. Periodontal treatment aims to address local and systemic factors fostering inflammation and tissue destruction, promote resolution of inflammation, and maintain tissue homeostasis. In periodontal tissues, the inflammatory microenvironment is governed by infiltration of myeloid cells where local stromal macrophages (Mφ) help to augment immune cell activation and prevent tissue damage. Mφ exhibit inherent functional plasticity initiating innate responses and shaping the adaptive arm of immunity to adeptly respond according to a myriad of pathogenic stimuli.^1^, ^2^, ^3^ The phagocytic abilities of both tissue‐resident Mφ and monocyte‐derived recruited Mφ are essential in innate defense as well as in the regulation of acquired immunity. Studies report differential transcriptional profiling in the tissue resident versus recruited Mφ derived from circulating monocytes, signifying that location and environmental triggers stimulate their polarization.^1^ Phenotypically polarized Mφ can differentiate into M1 (classical) or M2 (alternative) phenotypes.^4^ Exemplifying opposing activities, M1 Mφ promote bacterial killing and a pro‐inflammatory environment by increased production of IL‐6, TNF‐α, inducible nitric oxide synthase (iNOS) among other pro‐inflammatory molecules, while M2 Mφ are anti‐inflammatory in nature favoring resolution of inflammation and wound‐healing including IL‐10, arginase (ARG), resolvins, and TGF‐β.

Prior studies from our lab and others have shown that the priming of naive monocytes to host cytokines, such as IFN‐γ or IL‐4, gives rise to different Mφ phenotypes.^5^, ^6^, ^7^ This has considerable consequences for how Mφ detect, phagocytose, and kill bacteria. Mφ polarization in pathologic conditions such as periodontitis most appropriately represents a continuum, as opposed to two distinct scenarios, involving heterogeneity of the effector cells to a spectrum of polarization states that do not fit to the oversimplified M1/M2 classification.^8^ Moreover, the local microenvironment dependent abundance of Mφ within tissues is finely controlled through M‐CSF and GM‐CSF signaling, enabling appropriate responses to the microbial challenge or repair following an injury.^2^, ^3^

Successful periodontal therapy and patient compliance results in reduction of bacterial load that may instigate a pro‐resolution environment favoring M2‐like Mφ polarization in humans, triggering Th2 responses such as immunotolerance, and tissue remodeling. This transition between M1‐like and M2‐like polarization can determine the pathogenesis of periodontal disease, given the dynamic interaction of immune mediated signaling governing the inflammatory component as well as tissue healing in the cyclical nature of periodontal disease. We hypothesized that nonsurgical periodontal therapy creates a M2‐like Mφ dominant microenvironment favoring resolution of periodontal inflammation. Previous studies have identified expression of M1‐like and M2‐like markers in human gingival tissues of patients with chronic periodontitis.^9^ However, to the best of our knowledge there has been no longitudinal study evaluating the influence of periodontal therapy on Mφ polarization. We anticipate that these changes can provide insightful findings on the role of pathogen stimulated immune responses in the development of periodontal diseases and conversely, the role of M2‐like Mφ in resolution of periodontal inflammation posttherapy.

## 2 MATERIALS AND METHODS

### 2.1 Subject selection and sample collection

Systemically healthy subjects aged 18–70 years presenting to the Postgraduate Periodontics Clinic at the College of Dentistry, University of Illinois Chicago, from March 2018 to May 2019 were recruited for this study. Based on sample size calculation, that was calculated a priori; the convenience sample from a multiethnic group of both sexes comprised 24 individuals, to determine significant differences in miRNA profiles at an overall familywise error rate (FWER) of *α* = .05 level. These 24 individuals were divided into 2 groups: periodontally healthy (Control, Healthy; *N* = 12) and Stage III, Grade B periodontitis (Experimental, Diseased; *N* = 12). Individuals were excluded if they presented with uncontrolled systemic disorders: hypertension, heart disease, bleeding disorders, were pregnant, diabetic, current smokers, or presented with oral pathologies other than periodontal disease. Subjects in the periodontally healthy group presented with probing depths (PD) ≤ 3 mm; no bleeding upon probing (BOP); no evidence of active clinical attachment loss, no evidence of radiographic alveolar bone loss, and ≥4 mm attached keratinized gingiva. Subjects with Stage III, Grade B Periodontitis presented with ≥4 teeth with ≥6 mm PD, ≥5 mm clinical attachment loss, radiographic alveolar bone loss extending to middle third of root and beyond, and ≥4 mm attached keratinized gingiva as we previously described.^10^, ^11^ All examinations were performed with a manual periodontal probe (PCPUNC‐15; Hu‐Friedy) by two investigators (J. R. U. and V. K.), with a high interexaminer correlation coefficient *κ* = 0.85.

For the diseased group, the first gingival biopsy was obtained at baseline (pretherapy) while a second biopsy was obtained 4–6 weeks after nonsurgical periodontal therapy (posttherapy) consisting of scaling and root planning and patient education of oral hygiene measures. The second preselected biopsy site was distinct from the first gingival biopsy to eliminate characterization of Mφ due to healing of the previously excised tissue. For the control group, samples were derived from healthy gingival tissues normally discarded during routine surgical crown lengthening procedures.

### 2.2 Total RNA isolation and RT‐qPCR

Tissue samples were lysed using the TissueLyzer and total RNA isolated using the miRNeasy kit (both from Qiagen) with the subsequent mRNA and miRNA expression analysis as we previously reported.^10^, ^11^ Expression levels of STAT1, STAT6, TGM2, CXCL10, CCL22, IL10, and normalization control GAPDH were examined by RT‐qPCR using gene specific primers (Sigma Aldrich) as previously described.^10^ Real‐time PCR based analysis was also used to assess the impact of therapy on bacterial gene products, including the gingipain, RgpA, and RNA polymerase subunit β from *Porphyromonas gingivalis* (Pg), and Elongation Factor Tu and Ribosomal protein L2 from *Aggregatibacter actinomycetemcomitans*, (Aa). The primers used for these experiments are listed in Table 1 (Integrated DNA Technologies).

**Table 1:** Primer sets used for human and murine M1, M2 macrophage markers and bacterial gene transcripts.

Source	Gene	Forward primer (5' → 3')	Reverse primer (5' → 3')
Human	STAT1	ACCCAATCCAGATGTCTATG	GAGCCTGATTAAATCTCTGG
	STAT6	TACTGAAGACTCAGACCAAG	GATGATTTCTCCAGTGCTTTC
	IL10	GCCTTTAATAAGCTCCAAGAG	ATCTTCATTGTCATGTAGGC
	TNF‐α	CCCTTTATTACCCCCTCCTTCA	ACTGTGCAGGCCACACATTC
	GAPDH	ACAGTTGCCATGTAGACC	TTTTTGGTTGAGCACAGG
Murine	COX2	ACTCATAGGAGAGACTATCAAG	GAGTGTGTTGAATTCAGAGG
	INOS2	CATCAACCAGTATTATGGCTC	TTTCCTTTGTTACAGCTTCC
	TGM2	CATACCTACAAGTACCCAGAG	CTTTCTCTGCCAGTTTGTTC
	ARG1	CTGACCTATGTGTCATTTGG	CATCTGGGAACTTTCCTTTC
*Porphyromonas gingivalis*	RgpA gingipain	AGTTCAATCCTGTAAAGAAC	TCTGCTGCGAGCACAACCTT
	RNA pol β	CAGTATGCTCAAGCGTAAGGA	CCAGGTAGTCAGTCTTACCA
*Aggregatibacter actinomycetemcomitans*	EF – Tu	GCAAATGGACGGTGCTATCT	CCCGGGAAGTCATATTGAGA
	Ribosomal protein L2	TACAGATCATTGCCCGTGAA	GCTTTACCCAATACGCGAAG

For mature miRNA‐155 quantification, miScript primers and miScript II RT Kits were purchased from Qiagen. One hundred nanograms total RNA was reverse‐transcribed according to manufacturer's instruction and reactions performed using miRNA specific primers, universal primer (Qiagen), and SYBR Green (Roche). RNU6B was used as endogenous control. Replicate *C*_t_ values were analyzed to calculate relative fold change using the ${\Delta \Delta }^{C_{t}}$ method and the normalized values plotted as histograms with standard deviations (SD).

### 2.3 Murine model of ligature‐induced periodontitis and its resolution

On Day 0, mice aged 8–12 weeks were randomly divided into two groups (*N* = 4/group), anesthetized using intraperitoneal injection of ketamine (87.5 mg/kg) and xylazine (12.5 mg/kg) cocktail, and periodontitis was induced using a 6.0 silk ligature placed bilaterally between the maxillary first and second molar. We used female mice for our study because they exhibit higher rate of disease progression in murine model of periodontitis.^12^ Ligatures were removed from all animals on Day 8 and one group of animals was euthanized. To examine the resolution of periodontal inflammation phase, the second group of mice was euthanized 10 days after removal of ligatures (Day 18). Mice with displaced ligature during the experimental period were excluded. Harvested gingival tissues were homogenized, total RNA extracted and expression levels of murine COX2, INOS2, TGM2, ARG1 were examined by RT‐qPCR using gene specific primers (Table 1; Sigma Aldrich) as described above. β‐actin served as normalization control.

### 2.4 Preparation of single cell suspension from murine gingiva and flow cytometric analysis

Murine gingiva (*n* = 4/group) was washed thrice with PBS supplemented with 2% FBS and 100 µg/mL penicillin/streptomycin and transferred into 2 mL tissue digestion solution containing 2 mg/mL collagenase IV and 1 mg/mL DNase I (Sigma Aldrich). Tissues were minced using a sterile surgical blade and incubated at 37°C for 1 h. Gingiva tissue digestion was stopped by adding 0.5 mM EDTA, homogenate filtered through a 70‐μm cell strainer and centrifuged at 800 rpm for 5 min. The supernatant was discarded, the cell pellet washed twice with 1 mg/mL DNase, and cells stained for specific antibodies for M1 and M2 Mφ. Briefly, cells were added to tubes containing 100 µL PBS supplemented with 1% (v/v) BSA and incubated on ice for 45 min with the following anti‐mouse antibodies (all from BioLegend): anti‐mouse F4/80 (PE antimouse F4/80 antibody, clone W20065B), anti‐CD80 (FITC anti‐mouse CD80 antibody, clone: 16‐10A1), and anti‐CD206 (Alexa Fluor® 647 anti‐mouse CD206 [MMR] antibody, clone: C068C2). Thereafter, cells were washed twice with PBS‐1% BSA and data acquired on a CytoFLEX Flow Cytometer (Beckman Coulter). FlowJo_v10.8.1 software (Tree Star) was used to analyze the flow cytometry data.

### 2.5 Statistical analysis

Data were analyzed using GraphPad Prism (GraphPad Software). Results were presented as ±SD or ±SEM from three independent replicates, and experiments were conducted at least three times. *p* values were calculated using a Student's *t* test and/or ANOVA for more than two groups. A *p* < .05 was considered significant. Age distribution in the healthy controls and disease groups was assessed by Mann–Whitney *U* test. Gender distribution across the groups was evaluated by McNemar *χ* ^2^ test.

## 3 RESULTS

### 3.1 Nonsurgical periodontal treatment improves clinical outcomes

The sample size comprised of 24 individuals. Accounting for the pretreatment and post‐treated sample collection in the diseased group, a total a of 36 biologic replicates were collected from the three groups as follows: Healthy Controls, *N* = 12; Diseased, pretherapy *N* = 12, and posttherapy *N* = 12. Periodontal clinical parameters and subject demographics are shown in Table 2. There were no statistically significant differences noted in age (*p* = .49) or gender (*p* = .55) across the groups in our cohort. The mean age of individuals in the healthy group was 43 ± 11.3 years (range: 32–60) while the mean age in the diseased group was 52 ± 9.31 years (range: 38–59). For individuals with Stage III, Grade B periodontitis, statistically significant reductions in PD, CAL, and BOP were observed posttherapy. Mean PD was 9 mm pretherapy (9.35 ± 1.28 mm), which was reduced to approximately 7 mm posttherapy (6.85 ± 0.89 mm). We also noticed significant gain in mean CAL (5.59 ± 0.35 mm vs. 9.35 ± 1.28 mm) and % BOP (25.0 ± 7.35 vs. 74 ± 4.89) posttherapy compared to pretherapy, respectively. For periodontally healthy subjects, mean PD was 2.75 mm, CAL and BOP values were 3.15 ± 0.12 mm and 7 ± 2.4, respectively.

**Table 2:** Subject demographics and clinical parameters.

Patient variables	Controls (*n* = 12)	Chronic periodontitis (*n* = 12)	Nonsurgical therapy (*n* = 12)	*p* Value
Age (years)	43 ± 11.13	52 ± 9.31		
Gender (M/F)	3/4	3/2		
Number of teeth present	28 ± 2.9	25 ± 3.7		<0.05
Mean PPD (mm)	2.75 ± 0.63	9 ± 1.52	6.85 ± 0.89	<0.001 <0.01 (posttherapy)
Mean CAL	3.15 ± 0.12	9.35 ± 1.28	5.59 ± 0.35	<0.001 <0.01 (posttherapy)
% BOP	7 ± 2.4	74 ± 4.89	25 ± 7.35	<0.001 <0.01 (posttherapy)

### 3.2 Reduced levels of periodontopathic bacterial transcripts in gingival biopsies posttherapy correlates with clinical improvements

**Figure 1:**
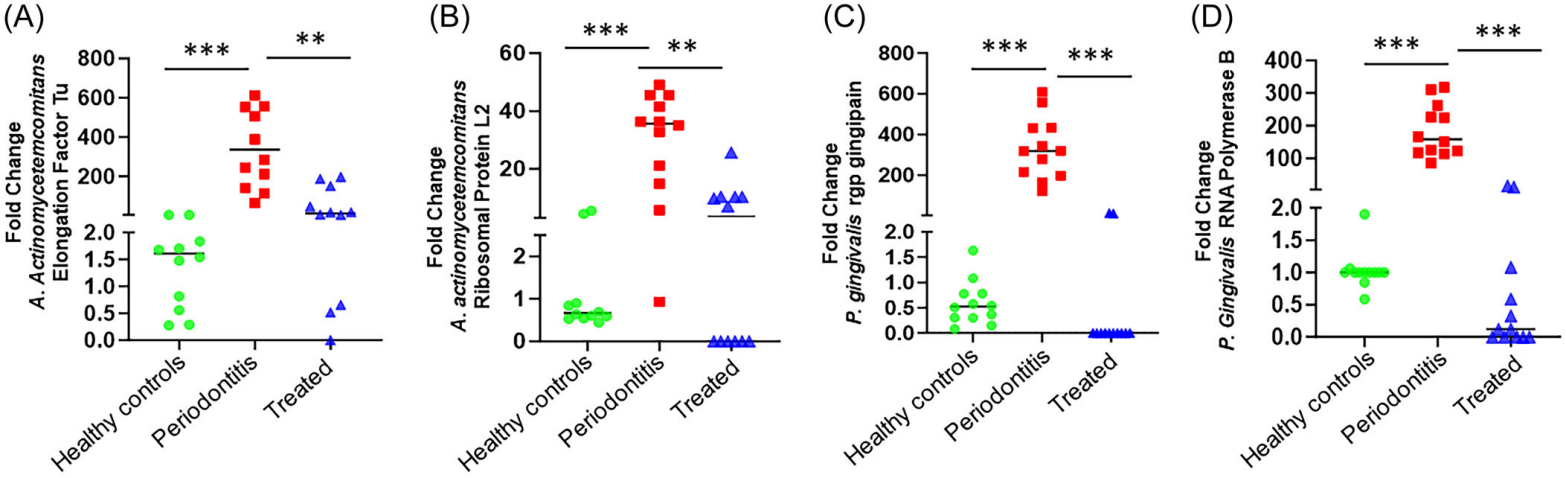
Reduced levels of Aa‐ and Pg‐encoded gene transcripts posttherapy. Quantitative RT‐PCR showing expression of Aa‐encoded (A) elongation factor‐Tu and (B) ribosomal protein L2 and Pg‐encoded (C) RgpA gingipain and (D) RNA polymerase subunit‐b in gingival biopsies after periodontal therapy. ANOVA was used to calculate *p* values. ***p < 0.001. Data is presented as ±SEM of all the detectable readings.

Significantly higher expression of Aa transcripts EF‐Tu (fold change: 378.02 ± 70.10; *p *< 0.0001) and RPL2 (fold change: 30.36 ± 4.62; *p *< 0.0001) (Figure 1A,B) and Pg transcripts for RgpA (fold change: 332.88 ± 43.92; *p *< 0.0001) and rpoB (fold change: 185.36 ± 23.20; *p *< 0.0001) (Figure 1C,D) and were noted in diseased pretherapy biopsy specimens compared to posttreatment samples. Both Pg and Aa gene transcripts were expressed at low levels or were undetectable in healthy control biopsies. Levels of the bacterial gene transcripts from gingival biopsies taken posttherapy approximated that seen in healthy controls. In posttherapy samples, Aa transcripts EF‐Tu (fold change: 51.79 ± 22.04, *p* < 0.001) and RPL2 (fold change: 6.11 ± 2.25; *p* < 0.001) were detected at low levels posttherapy. Pg transcripts for RgpA (fold change: 2.14 ± 1.45; *p* < 0.0001) and rpoB (fold change: 2.46 ± 1.53, *p* < 0.0001) showed significantly reduced expression levels or were not detected posttherapy.

### 3.3 M1‐like macrophage markers are downregulated after periodontal therapy

Macrophages display phenotype plasticity that allows them to exert unique functions depending on inflammatory status. Mφ phenotype can be broadly classified as classically (M1) or alternatively (M2) activated. M1 Mφ are primarily involved in Th1 responses, lymphokine production, and degradation of intracellular pathogens, whereas M2 Mφ trigger Th2 responses, immunotolerance, and tissue remodeling.^2^, ^4^, ^13^ Unique surface and intracellular markers can differentiate Mφ phenotype. Typically, M1 macrophages express CD68 and major histocompatibility complex‐II (MHC‐II), while CD163 and CD206 are the markers for the M2 macrophages. Additionally, M1 macrophages secrete pro‐inflammatory mediators including STAT1, TNF‐α, IL‐6, miR‐155, iNOS, and so forth.

**Figure 2:**
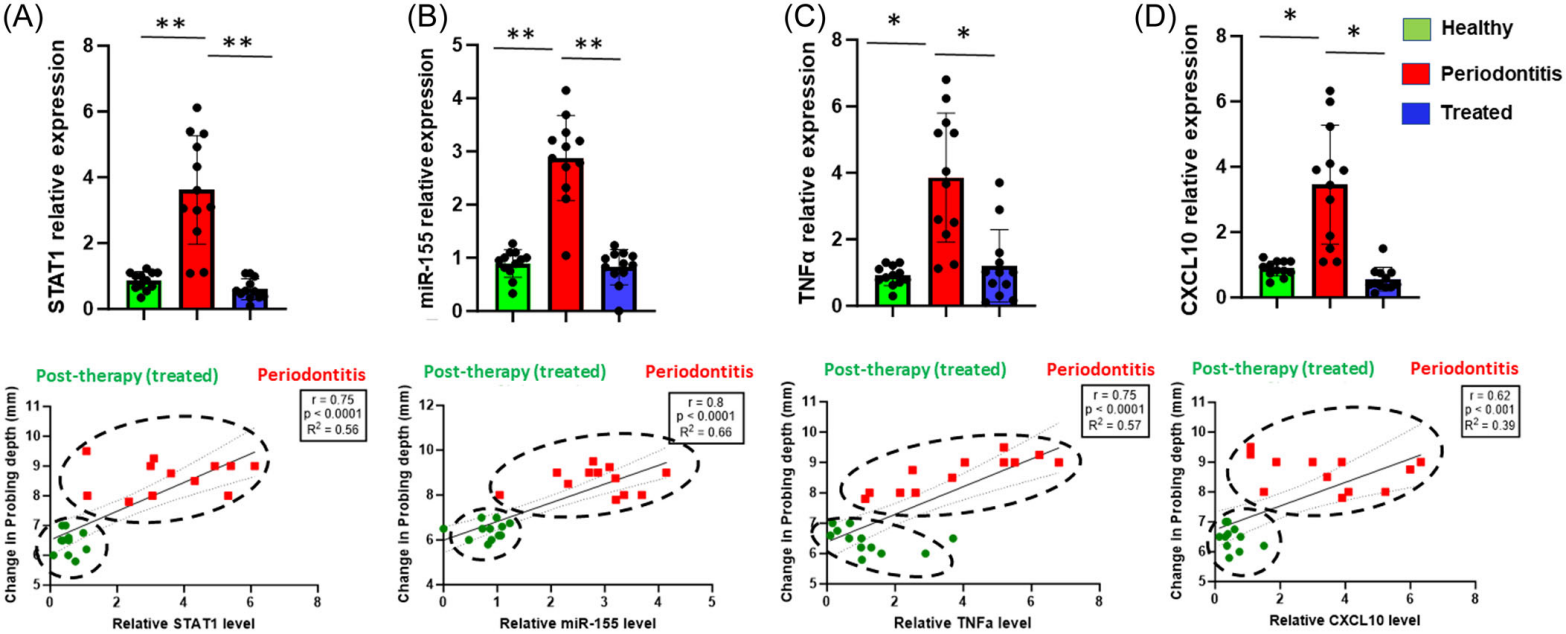
Downregulation of gingival M1 macrophage markers after nonsurgical therapy. Histograms showing relative expression of pro‐inflammatory (A) STAT1, (B) miR‐155, (C) TNF‐α, and (D) CXCL10 transcripts by quantitative PCR analysis in gingival biopsy samples of periodontitis patients before (*n* = 12) and after therapy (*n* = 12) as compared to those of healthy controls (*n* = 12). Results are normalized to those of controls and are represented relative to expression of GAPDH and RNU6, respectively. ANOVA was used to calculate *p* values. *p < .05, **p < .01. Data is presented as ±SEM of all samples. Lower panel: Pearson's correlation analysis of STAT1, miR‐155, TNF‐α and CXCL10 expression positively correlates with periodontal probing depth as measured in mm, in the periodontitis groups (pre‐ and posttherapy).

To investigate whether nonsurgical periodontal therapy can impact Mφ polarization, we evaluated changes in expression of the well characterized M1‐like markers, STAT1 and miR‐155.^14^, ^15^ We compared expression of these markers in gingival biopsies derived from periodontally healthy and diseased (pre‐/posttherapy) subjects. We observed significantly higher expression of STAT1 (Fold change ~4, *p *< .01) and miR‐155 (Fold change ~3, *p *< .01) in diseased gingival samples compared to healthy controls (Figure 2A,B). Conversely, expression levels of STAT1 and miR‐155 in gingival biopsies taken posttherapy were significantly decreased (*p *< .01) to levels observed in the healthy cohort. Likewise, upon assessing M1‐like related pro‐inflammatory cytokine/chemokine expression, we found higher expression of TNF‐α (Fold change ~4, *p *< .05) and CXCL10 (Fold change ~3.5, *p *< .05) in diseased samples at baseline compared to healthy controls (Figure 2C,D). Expression of both pro‐inflammatory genes were significantly reduced in posttreatment biopsies (*p* < .05). These results clearly show that M1‐like Mφ markers are downregulated in gingiva from periodontally diseased subjects after nonsurgical periodontal therapy.

We further investigated whether the expression of M1 markers is responsive to periodontal therapy. Interestingly, higher levels of all the M1 markers STAT1 (*r* = .75; *p *< 0.0001), miR‐155 (*r* = .8; *p *< 0.0001), TNF‐α (*r* = .75; *p *< 0.0001), and CXCL10 (*r* = .62; *p *< 0.001) correlated with PPD in the periodontitis group (Figure 2; lower panel). The expression of M1 markers also showed a significantly inverse correlation between the periodontitis and post‐therapy groups. Overall, these findings strongly support that M1‐like Mφ marker expression respond to periodontal therapy and correlate with clinical parameters.

### 3.4 M2‐like macrophage markers are upregulated after periodontal therapy

**Figure 3:**
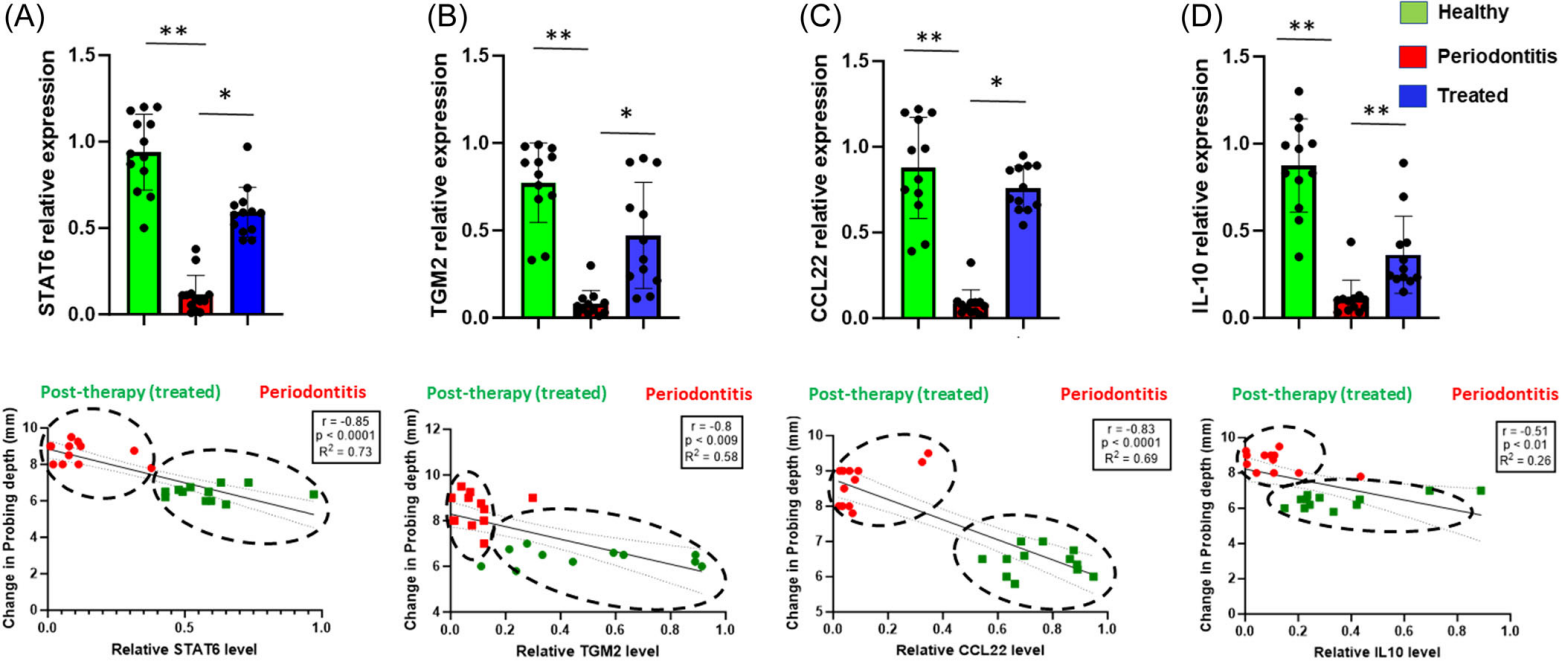
M2 macrophage markers are upregulated in gingiva after nonsurgical therapy. Histograms showing relative expression of anti‐inflammatory markers (A) STAT6, (B) TGM2, (C) IL10, and (D) CCL22 by quantitative RT‐PCR analysis in gingival biopsy samples of periodontitis patients before (*n* = 12) and after therapy (*n* = 12) as compared to those of healthy controls (*n* = 12). Results are normalized to those of controls and are represented relative to expression of GAPDH. Student's *t* test was used to calculate *p* values. *p < .05, **p < .01. Data is presented as ±SEM of *n* = 12/group. Lower panel: Pearson's correlation analysis of STAT6, TGM2, IL10, and CCL22 expression negatively correlates with periodontal probing depth as measured in mm, in the periodontitis groups (pre‐ and posttherapy).

The antagonistic expression levels of M2‐like Mφ activation markers in response to periodontal therapy was evaluated by expression profiles of TGM2 and STAT6.^14^, ^16^ We also examined expression of M2‐like associated effector chemokines/cytokines (CCL22, IL10) in response to therapy. In contrast to the elevated expression of M1 markers observed, we found diminished expression of M2‐like markers STAT6 (Fold change ~10, *p *< .01) and TGM2 (Fold change ~8, *p *< .01) in diseased specimens compared to healthy controls (Figure 3A,B). Conversely, periodontal therapy rescued STAT6 (Fold change ~4, *p *< .05) and TGM2 (Fold change ~3.5, *p *< .05) expression. Likewise, expression of M2‐like associated anti‐inflammatory cytokines IL10 (Fold change ~8, *p *< .01), and CCL22 (Fold change ~8, *p *< .01) were significantly lower in the pretreatment diseased biopsies, levels of which were significantly increased in posttherapy samples (IL10, fold change ~2, *p *< .05; CCL22, fold change ~6, *p *< .01, Figure 3C,D).

Next, we examined whether M2‐like markers correlate with disease and respond to therapy. Our correlation analysis showed that M2‐like markers STAT6 (*r* = −.85; *p *< 0.0001), TGM2 (*r* = −.8; *p *< 0.009), CCL22 (*r* = −.85; *p *< 0.0001), and IL‐10 (*r* = −.51; *p *< .01) exhibit significant negative correlation with PPD (Figure 3; lower panel). Collectively, these results demonstrate that M2‐like Mφ or the repair‐associated markers in gingival biopsies from diseased biopsies are responsive to nonsurgical periodontal therapy.

### 3.5 Murine macrophages exhibit dynamic changes in phenotype markers during ligature‐induced periodontitis and its resolution

**Figure 4:**
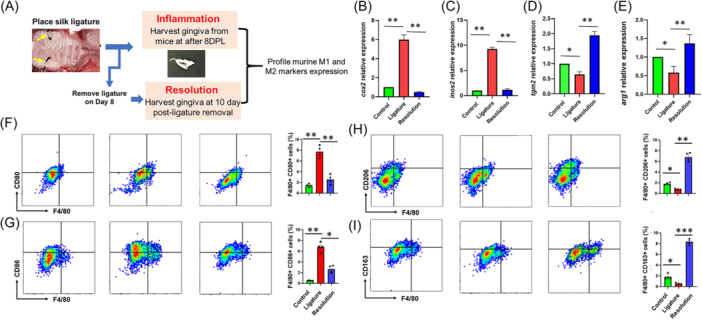
M1 and M2 macrophage markers exhibit antagonistic expression in murine ligature‐induced periodontitis and resolution model. (A) Schematic showing murine periodontal inflammation and its resolution model. Total RNA was isolated from healthy and inflamed gingival biopsies from mice (*n* = 4/group) subjected to ligature‐induced periodontitis for 8 days and after 10 days postligature removal. Expression of M1 and M2 markers was quantified by RT‐qPCR. Histograms showing relative fold change expression of (B) *cox‐2*, (C) *iNOS2*, (D) *tgm2*, and (E) *arg1* in murine gingiva. Transcript expression was normalized to β‐actin. Proportion of (F and G) M1‐like and (H and I) M2‐like Mφ in murine gingival tissues during periodontal inflammation and its resolution. Gingival cells were stained with F4/80 and CD80 or CD86 to identify F4/80+CD80+ and F4/80+CD86+ M1 Mφ, while F4/80 in combination with CD206 or CD163 was used to elucidate the proportion of F4/80+CD206+ and F4/80+CD163+ M2 Mφ in the gingival tissues (F4/80+CD206+). Left panel shows the representative scattered plots, and right panel exhibits the total percentage of M1 and M2 Mφ. FlowJo_v_10.9.0 was used to generate scattered plot and evaluate the percentage of M1/M2 Mφ. Student's *t* test was used to calculate *p* values. *p < .05, **p < .01. Data is presented as ±SEM of *n* = 4/group.

Our human studies suggest that M1‐like and M2‐like Mφ phenotypes exhibit dynamic changes pre‐ and posttherapy. To confirm these findings, we examined Mφ marker expression in gingival biopsies collected from mice subjected to ligature‐induced periodontitis. Placement of ligature alone causes dysbiosis and induces periodontal inflammation and marked alveolar bone loss. For resolution, we removed the ligature after periodontal disease was established (Day 8) in a parallel animal cohort to activate the resolution of inflammation phase process for an additional 10 days (DPLR). (Figure 4A). We observed significantly higher expression of murine M1 inflammatory markers *cox2* (Fold change ~6) and *inos2* (Fold change ~8) in the gingiva at 8 days of ligature placement, which marks disease establishment (Figure 4B,C). Ten days after ligature removal (Day 18), we noted significantly less *cox2* (Fold change ~6) and *inos2* (Fold change ~8) compared to Day 8 demonstrating expression levels similar to no ligature control. These results suggest a reversal in Mφ phenotype after removal of inflammatory stimuli.

Next, we determined if M2 Mφ markers in our ligature‐induced periodontitis murine model exhibit a pattern of expression similar to that observed in pre‐/posttherapy human gingival samples. Our results show significantly reduced levels of transglutaminase 2 (*tgm2;* Fold change ~0.75) and arginase1 (*arg1;* Fold change ~0.5) in gingiva 8 days after ligature placement compared to animals without ligature (Figure 4D,E). Ten days after ligature removal (Day 18) there was a marked increase in both *tgm2* (fold change ~2.5) and *arg1* (fold change ~2) levels compared to 8 days indicating a pronounced activation of pro‐resolution M2 phenotype. Overall, these results strongly support that M1 and M2 phenotypic markers in human as well as murine gingiva exhibit antagonistic expression profiles during both the inflammatory and resolution phases of inflammation indicating a functional role of Mφ polarization in periodontal tissue immune homeostasis.

To validate our RT‐qPCR results, we examined gingival M1 and M2 murine Mφ by flow cytometric analysis. Single cell suspensions were isolated from gingiva from no ligature control mice, mice subjected to ligature placement (inflammatory phase, Day 8), and ligature removal (resolution phase, Day 10 postligature removal [DPLR]). Our results show marked increase in M1 phenotype markers F4/80+CD80+ (7.1 ± 1.6%) and F4/80+CD86+ (6.7 ± 0.9%) after Day 8 compared to controls. The expression of these markers reversed significantly 10 days after removal of ligature (F4/80+CD80+ : 2.5 ± 0.9% and F4/80+CD86+ : 2.6 ± 0.7%) compared to no ligature control (F4/80+CD80+ : 1.4 ± 0.3%; F4/80+CD86+ :0.5 ± 0.05%; Figure 4F,G). Conversely, M2 Mφ increased significantly as observed higher F4/80+CD206+ (6.8 ± 0.8%; *p *< 0.001) and F4/80+CD163+ (8.3 ± 0.5%; *p *< 0.001) cells following induction of resolution (after ligature removal) compared to Day 8 mice (F4/80+CD206+ : 0.8 ± 0.1% and F4/80+CD163+ : 0.5 ± 0.2%) or control mice (F4/80+CD206 + : 1.8 ± 0.2%; F4/80+CD163+ : 1.9 ± 0.5; Figure 4H,I). These results indicate a dynamic polarization of gingival Mφ toward M2 phenotype upon removal of the dysbiotic trigger (i.e., ligature).

## 4 DISCUSSION

Periodontitis is a polymicrobial, inflammatory disease that commences with periodontal pathogen‐induced immune responses, affecting the supporting tissues of the teeth. The chronicity of periodontal inflammation has been characterized as an aggregated set of events highlighted by a continuous pro‐inflammatory environment accompanied with cyclic bursts of tissue healing patterns, including cell apoptosis, wound healing, cell proliferation, and tissue repair.^17^ Importantly, periodontal inflammation is largely governed by infiltration of myeloid cells, particularly Mφ. Based on the concept of immuno‐modulation, Mφ play important roles as effector cells in mediating Th1 driven and Th2 derived immune responses. Recent evidence suggests a profound role of Mφ polarization in gingival tissue from subjects with chronic periodontitis.^8^, ^15^, ^18^ However, to the best of our knowledge there has been no study undertaken to longitudinally characterize Mφ polarization after periodontal therapy in humans.

Nonsurgical periodontal therapy aims to remove the etiological agents of periodontal inflammation and in doing so, fosters changes in the cellular milieu and microenvironment supportive of resolution of inflammation. Reduction in Pg and Aa bacterial loads quantified as reduced periopathogen encoded transcripts accompanied improvements in clinical parameters observed. However, it should be noted that this is not to be interpreted as a treatment endpoint as healing is often incomplete in patients with Stage III, Grade B periodontitis and thus may require surgical intervention to address residual deep PD and osseous defects. The intent of this experimental design was to initiate the resolution phase of inflammation and the beginning of healing to observe shifts in M1/M2 phenotype. We demonstrated that posttherapy changes occur in M1/M2 Mφ polarization markers strongly suggesting a role of Mφ in PD pathogenesis and during resolution phase of periodontal inflammation. The increased levels of M1 markers and pro‐inflammatory cytokines observed in disease are in concordance with other reports evaluating the role of Mφ polarization.^19^, ^20^, ^21^ Bonafide evidence of in vivo Mφ alternative activation in the resolution phase, which elicits a strong Th2 response, was observed by increased IL10 and CCL22 expression, in a more comprehensive functional perspective after nonsurgical periodontal therapy. CCL22, also known as macrophage‐derived chemokine (MDC) is mainly produced by Mφ upon the stimulation with microbial products and upregulated by Th2‐type cytokines to enhance the chemotactic migration and recruitment of DCs and Th2 cells.^22^ In addition to the host immune responses, environmental and epigenetic factors (miRNAs) are also responsible in directing the fate of Mφ M1‐M2 switch in periodontal disease.^13^, ^23^, ^24^ While evaluating the gene expression profiles in a study using nonhuman primate model for periodontitis, Gonzalez and colleagues, noted remarkable differences in the distribution of gene signatures for M0, M1, and M2 macrophages in healthy tissues with the younger animals during initiation, progression, and resolution of periodontitis.^25^ In this study, young animals demonstrated a dramatic increase in M1 genes (PAMPs, TLR2, and TLR4, etc.), which remained increased compared to healthy levels during the disease process. Conversely, the levels of M2 macrophage genes (CD63, CD32, etc.) only found to be elevated in young animals during resolution.^25^ Therefore, genes specific to macrophage polarization reflect upon gingival health, disease, and resolution of periodontitis.

In congruence with previous studies, we noted higher expression of M1 and lower expression of M2 markers in inflamed human gingiva, which improved posttherapy suggesting dynamic molecular changes in immune cell polarization and activity after 4–6‐week of treatment. Monitoring these molecular profiles suggests a new approach in assessing the success of periodontal treatment. For instance, in this study, M1 associated markers STAT1, miR‐155, or the effector cytokines/chemokines TNF‐α and CXCL10 were responsive to periodontal therapy and may be used to target a specific subset of patients with an exaggerated immune response. Our data corroborates with the earlier published reports demonstrating a role of higher expression and activation of STAT1 in periodontitis patients.^26^, ^27^, ^28^ Wei and colleagues showed that activation of STAT1 pathway was linked with endothelial nitric oxide synthase gene knockout‐related (Nos3‐/‐) mouse model of periodontitis. Treatment with STAT1 inhibitor results in amelioration of bone resorption and periodontal destruction in periodontitis lesions in vivo.^28^ Interestingly, various studies demonstrated TNF‐α‐induced CXCL10 synergistically contribute to the onset and progression of periodontitis.^29^, ^30^, ^31^ These cytokines are involved in chemoattraction of macrophages, T cells, NK cells, and dendritic cells.^32^ Indeed salivary expression of pro‐inflammatory markers TNF‐α, sTNF‐R1, and sTNF‐R2 correlates with the early stages of periodontal inflammation.^33^ Our findings corroborate with previous reports highlighting a positive correlation of higher inflammatory mediators (involved in M1 phenotype) with periodontal disease progression. Conversely, M2‐associated markers STAT6, TGM2 or the effector cytokines/chemokines IL10 and CCL22 were elevated after therapy and may have utility in evaluating response to therapy. IL‐4/IL‐13 mediated activation of the JAK3/STAT6 signaling pathway play an important role in the phenotypic polarization of M2A macrophages and tissue repair.^34^ STAT6 is not sufficient to induce M2 alone, however, other factors viz IL4‐R or IL‐10 are also required for the M2 macrophage polarization and wound healing in mice.^35^ Interestingly, increment of IL‐10 levels in the gingival crevicular fluid correlates with the reduced bone‐resorptive activity.^36^ In conjunction with IL‐10, IL‐4/CCL22/CCR4 axis increases the regulatory T‐cell migration and concomitantly suppresses inflammatory bone loss in murine experimental periodontitis while TGM2 contributes towards stabilization of the extracellular matrix and wound healing in periodontium rather than gingival inflammation.^37^, ^38^ Overall, M1 and M2 Mφ play a critical role in the overall immune homeostasis by actively releasing a network of pro‐ and anti‐inflammatory mediators that control periodontal inflammation and its resolution. Our results unequivocally showed that periodontal therapy can shape macrophage polarization by reducing the burden of local initiators of inflammation as evident by reduced M1 and a concomitant increase in M2 markers.

These findings were further validated in our murine ligature‐induced periodontitis and resolution model. M1‐like Mφ were reduced significantly with a concomitant increase in M2‐like Mφ postligature removal. Our results demonstrate that Mφ are highly responsive to the periodontal microenvironment and swiftly shift toward a reparative M2 phenotype upon removal of dysbiotic triggers. That is, a decrease in levels of M1 Mφ markers and conversely, increase in M2 Mφ polarization markers correlate with clinical improvement in periodontal inflammation.

Our results highlight the significant role for immune governed mechanisms in Mφ plasticity and polarization in periodontal inflammation. An innovative and noteworthy finding was the restoration of M2 marker expression levels posttherapy to similar levels observed in periodontally healthy subjects, as well as reduction in the M1 marker expression levels that coincided with clinical improvement. This further affirms the immune mediated restoration and/or maintenance of periodontal tissue homeostasis. It is the inherent plasticity of Mφ that adeptly changes according to the tissue microenvironment that determines M1 to M2 switch and vice versa. This Mφ plasticity is essential to initiate pathogen/antigen clearance as well as instigate tissue repair.^39^, ^40^

In conclusion, this is one of the first translational studies to longitudinally assess the M1/M2 Mφ profiles before and after therapy in periodontitis subjects. Extensive functional experiments in vitro, in animal models, along with studies with larger cohorts in humans, are required to validate the suitability of examining the M1/M2 Mφ axis in periodontal disease and resolution phases, for the development of potential biomarkers and therapeutic targets for periodontitis. Our findings highlight the immuno‐modulatory role of M1/M2 Mφ polarization in periodontal disease pathogenesis and likewise, the impact of periodontal therapy in fostering a switch towards the M2 Mφ phenotype. Assessment of M2 Mφ markers may provide clinically relevant information to evaluate patient response to periodontal therapy and may be useful to target nonresponders with exaggerated immune responses.

## AUTHOR CONTRIBUTIONS

All authors have made substantial contributions to conception, design of the study, and given final approval of the version to be published. Juhi R. Uttamani and Varun Kulkarni were calibrated and performed clinical data measurements, periodontal therapy as well as obtained biomaterials from the patients. Juhi R. Uttamani, Varun Kulkarni, Rafsar Ali Naqvi, Araceli Valverde, and Afsar R. Naqvi were involved in data collection and data analysis. Juhi R. Uttamani, Varun Kulkarni, Thomas Van Dyke, Salvador Nares, and Afsar R. Naqvi were involved in data interpretation, and drafting the manuscript.

## CONFLICT OF INTEREST STATEMENT

The authors declare no conflict of interest.

## ETHICS STATEMENT

The prospective longitudinal case–control pilot study was approved by the Institutional Review Board and the Ethics Research Committee at the University of Illinois Chicago, College of Dentistry (IRB Protocol# 2017‐1064). The protocol and procedures were ethically reviewed and approved by the Institutional Animal Care and Use Committee at the University of Illinois Chicago (ACC # 22‐122). All experiments were performed in accordance with institutional and national guidelines for the care and use of laboratory animals.

## Data Availability

The data that support the findings of this study are available on request from the corresponding author. The data are not publicly available due to privacy or ethical restrictions.
